# Supplementary figures and images for: A study of autoencoders as a feature extraction technique for spike sorting
Source: PLoS One. 2023 Mar 9;18(3):e0282810. doi: 10.1371/journal.pone.0282810 (PMC9997908; doi:10.1371/journal.pone.0282810)

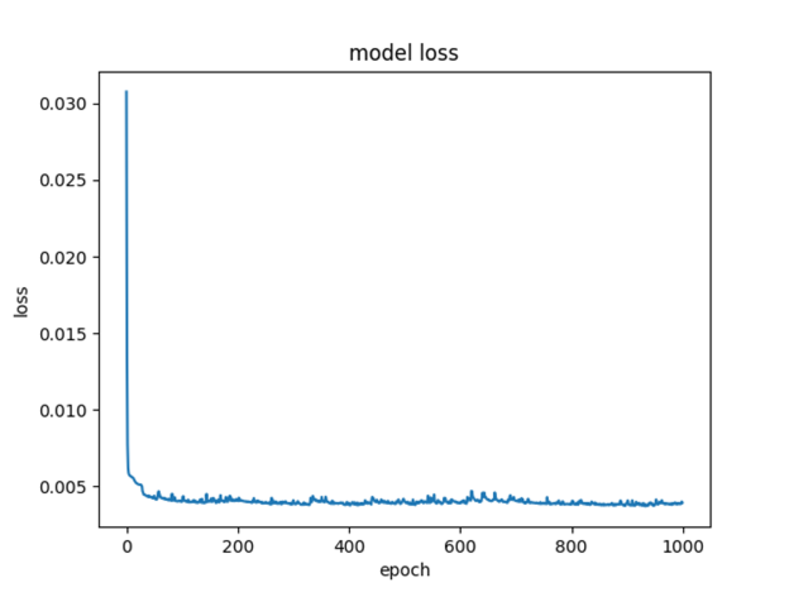

Supplement: S1 Fig — Loss values during the training of the model on Sim4 for an AE model. (TIF) [file pone.0282810.s001.tif]

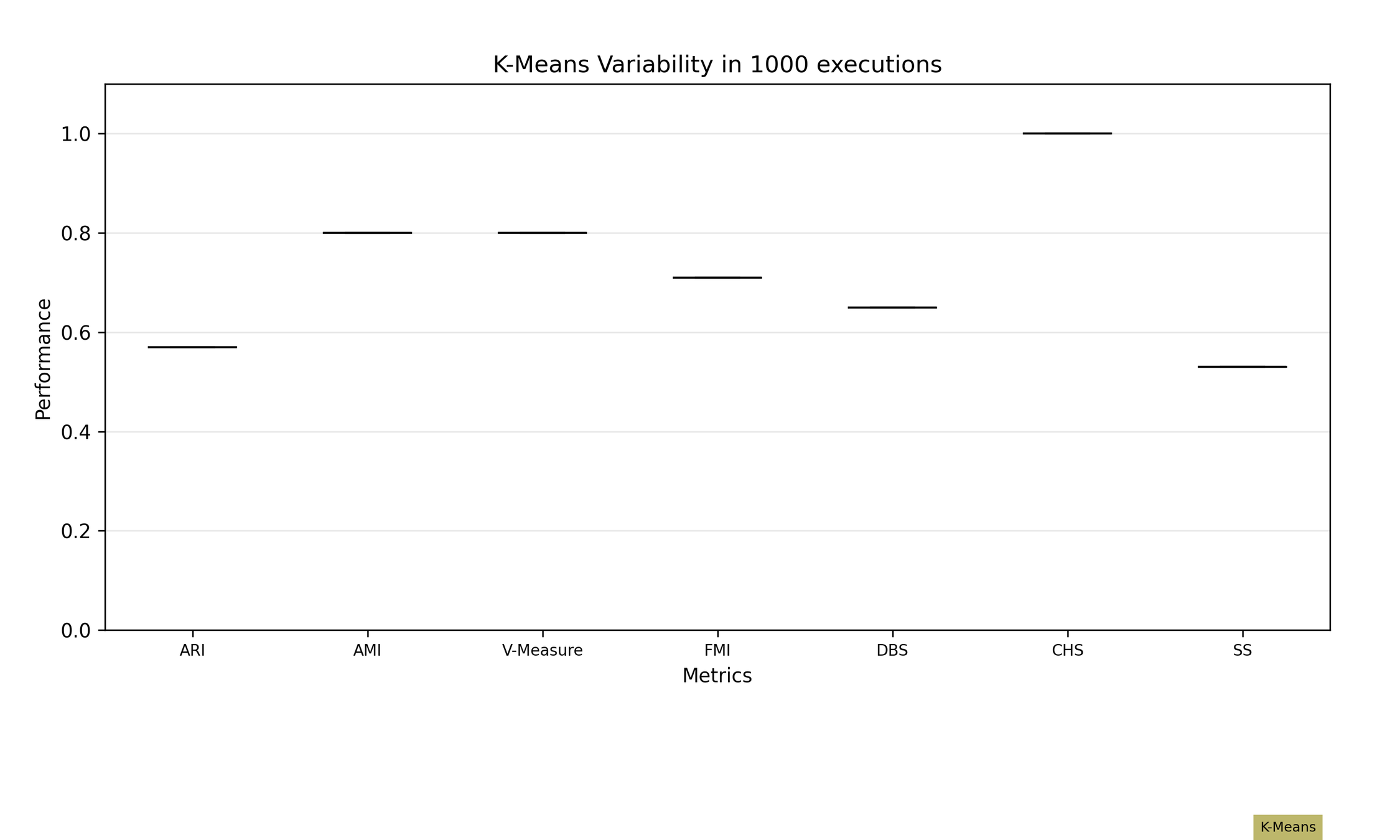

Supplement: S2 Fig — Variability of the used K-Means implementation for 1000 executions on the features provided by PCA using k-means++ initialization. (TIF) [file pone.0282810.s002.tif]

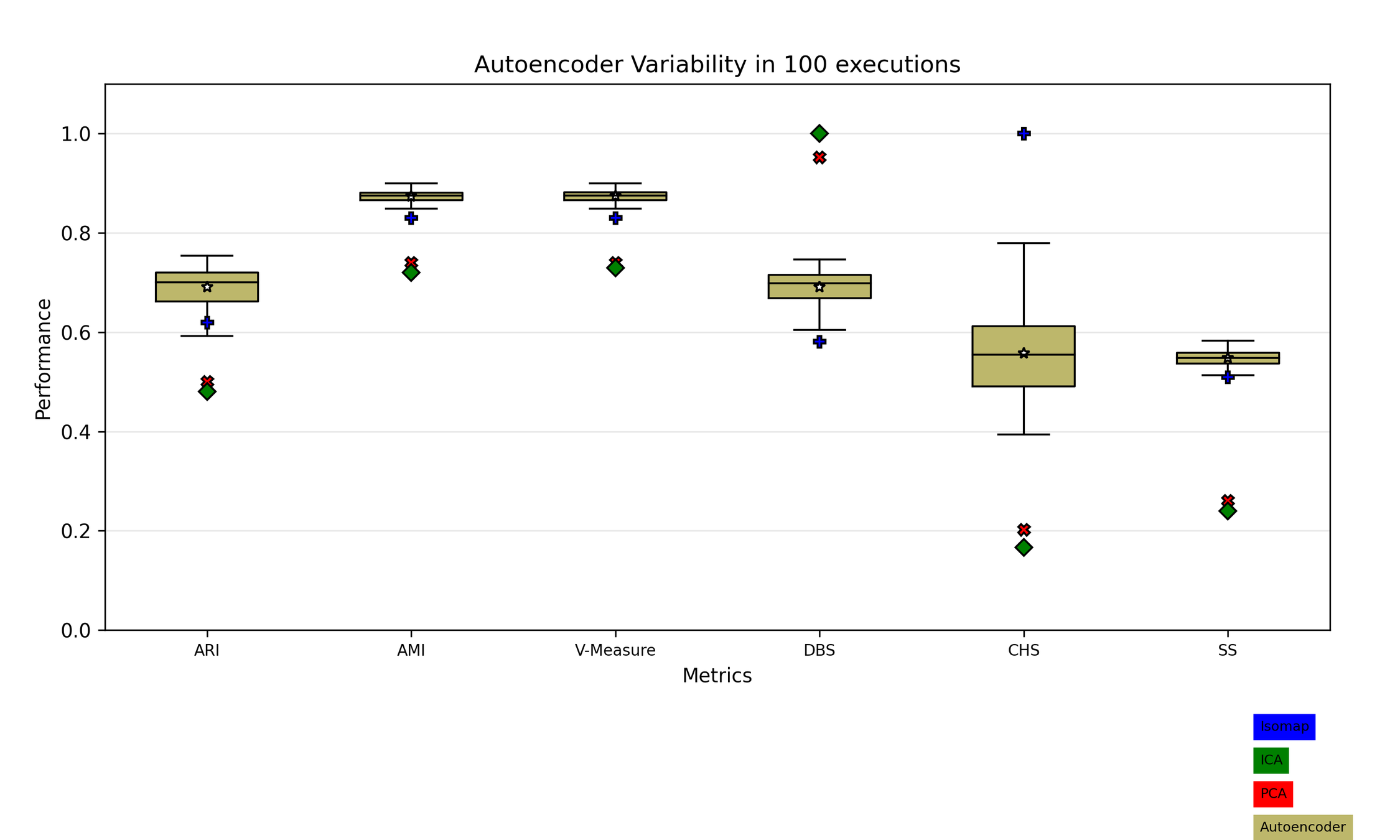

Supplement: S3 Fig — Variability of the shallow autoencoder on the Sim1 dataset (17 clusters) over 100 executions, where the white star represents the average score over 100. An important note is that DBS has lower scores for a higher performance and as DBS and CHS have only a lower bound they have been scaled between 0 and 1 by dividing with the maximum value across all executions of the autoencoder and the other methods. (TIF) [file pone.0282810.s003.tif]

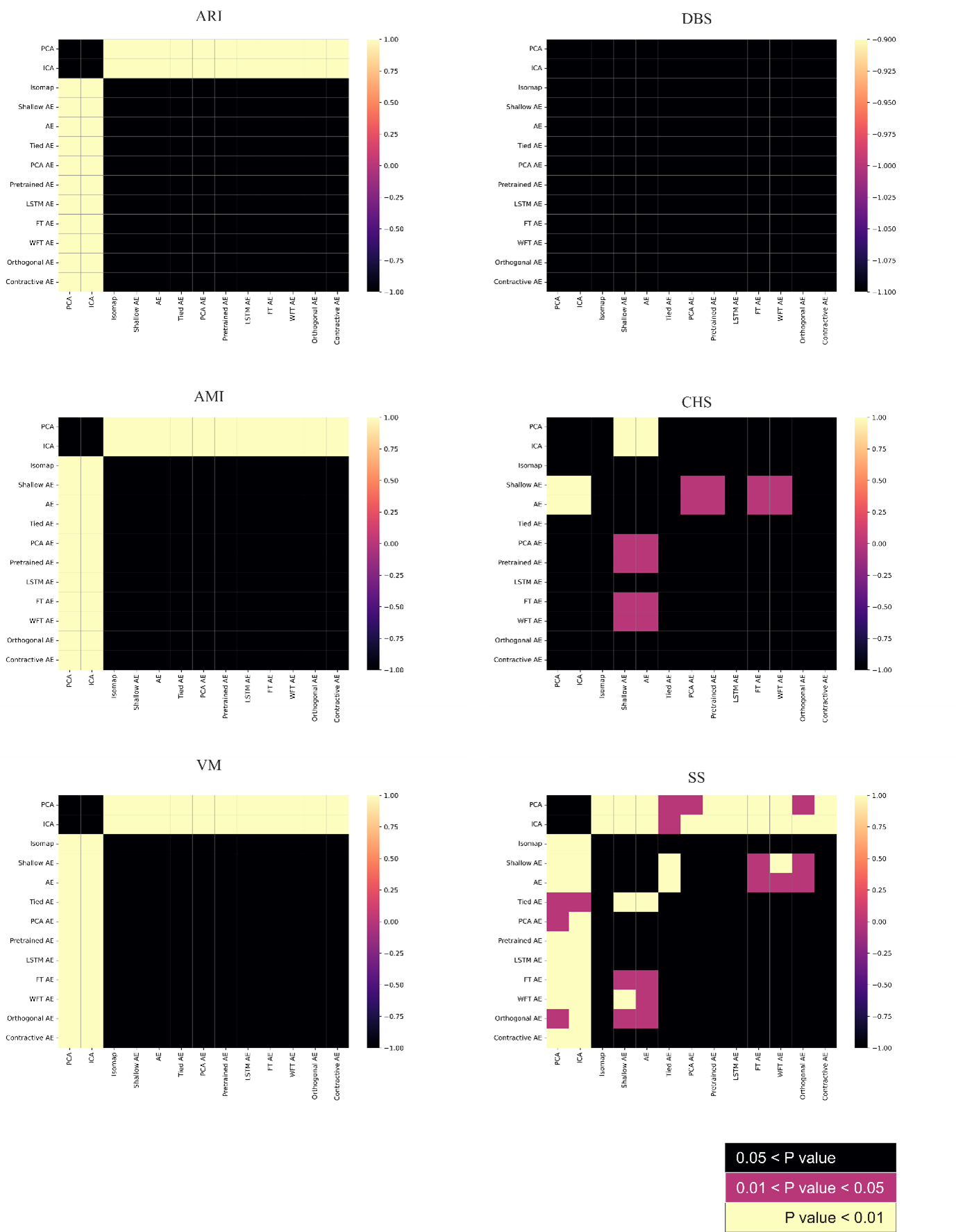

Supplement: S4 Fig — P value of t-tests (with a Bonferroni correction) for each of the metric on all 95 simulations. (TIF) [file pone.0282810.s004.tif]
