# Supplementary material for: A study of autoencoders as a feature extraction technique for spike sorting
Source: PLoS One. 2023 Mar 9;18(3):e0282810. doi: 10.1371/journal.pone.0282810 (PMC9997908; doi:10.1371/journal.pone.0282810)
Supplement: S1 Table — Borda rank aggregation of results for each metric on the 4 synthetic datasets (simulations 1, 4, 16, 35). (DOCX) [file pone.0282810.s005.docx]

| Rank | ARI | AMI | VM | DBS | CHS | SS |
| --- | --- | --- | --- | --- | --- | --- |
| 1 | Shallow AE | Shallow AE | Shallow AE | Shallow AE | Isomap | Contractive AE |
| 2 | AE | AE | AE | Isomap | Contractive AE | Shallow AE |
| 3 | Contractive AE | Contractive AE | Contractive AE | Pretrained AE | Shallow AE | Isomap |
| 4 | Pretrained AE | Pretrained AE | Pretrained AE | AE | AE | AE |
| 5 | Isomap | Isomap | Isomap | Contractive AE | LSTM AE | Pretrained AE |
| 6 | Tied AE | LSTM AE | LSTM AE | PCA | Pretrained AE | Tied AE |
| 7 | LSTM AE | Tied AE | Tied AE | ICA | PCA | LSTM AE |
| 8 | PCA AE | PCA | PCA | LSTM AE | ICA | PCA |
| 9 | ICA | ICA | ICA | PCA AE | WFT AE | ICA |
| 10 | PCA | PCA AE | PCA AE | FT AE | PCA AE | PCA AE |
| 11 | WFT AE | WFT AE | WFT AE | Tied AE | FT AE | WFT AE |
| 12 | Orthogonal AE | FT AE | FT AE | WFT AE | Tied AE | FT AE |
| 13 | FT AE | Orthogonal AE | Orthogonal AE | Orthogonal AE | Orthogonal AE | Orthogonal AE |
