# Supplementary material for: A study of autoencoders as a feature extraction technique for spike sorting
Source: PLoS One. 2023 Mar 9;18(3):e0282810. doi: 10.1371/journal.pone.0282810 (PMC9997908; doi:10.1371/journal.pone.0282810)
Supplement: S2 Table — Borda rank aggregation of the results for each metric on all 95 synthetic datasets. (DOCX) [file pone.0282810.s006.docx]

| Rank | ARI | AMI | VM | DBS | CHS | SS |
| --- | --- | --- | --- | --- | --- | --- |
| 1 | AE | Shallow AE | Shallow AE | AE | AE | Shallow AE |
| 2 | Shallow AE | AE | AE | Shallow AE | Shallow AE | AE |
| 3 | Isomap | Isomap | Isomap | ICA | PCA | LSTM AE |
| 4 | LSTM AE | LSTM AE | LSTM AE | Pretrained AE | LSTM AE | Pretrained AE |
| 5 | PCA AE | Pretrained AE | Pretrained AE | Contractive AE | Orthogonal AE | Isomap |
| 6 | Pretrained AE | WFT AE | WFT AE | PCA | Isomap | PCA AE |
| 7 | Tied AE | Tied AE | Tied AE | LSTM AE | Pretrained AE | WFT AE |
| 8 | Contractive AE | PCA AE | PCA AE | FT AE | ICA | Orthogonal AE |
| 9 | FT AE | Contractive AE | Contractive AE | PCA AE | WFT AE | FT AE |
| 10 | Orthogonal AE | Orthogonal AE | Orthogonal AE | Orthogonal AE | PCA AE | Tied AE |
| 11 | WFT AE | FT AE | FT AE | Tied AE | FT AE | Contractive AE |
| 12 | PCA | PCA | PCA | WFT AE | Contractive AE | PCA |
| 13 | ICA | ICA | ICA | Isomap | Tied AE | ICA |
