# Supplementary material for: A study of autoencoders as a feature extraction technique for spike sorting
Source: PLoS One. 2023 Mar 9;18(3):e0282810. doi: 10.1371/journal.pone.0282810 (PMC9997908; doi:10.1371/journal.pone.0282810)
Supplement: S3 Table — Borda rank aggregation of the results for each metric on real data. (DOCX) [file pone.0282810.s007.docx]

| Rank | DBS | CHS | SS |
| --- | --- | --- | --- |
| 1 | AE | AE | AE |
| 2 | Contractive AE | Isomap | Tied AE |
| 3 | Isomap | Contractive AE | Isomap |
| 4 | Tied AE | Tied AE | PCA |
| 5 | Orthogonal AE | PCA AE | Shallow AE |
| 6 | PCA AE | Pretrained AE | Contractive AE |
| 7 | Pretrained AE | Shallow AE | PCA AE |
| 8 | Shallow AE | PCA | Pretrained AE |
| 9 | ICA | Orthogonal AE | Orthogonal AE |
| 10 | PCA | FT AE | ICA |
| 11 | FT AE | LSTM AE | FT AE |
| 12 | LSTM AE | ICA | LSTM AE |
| 13 | WFT AE | WFT AE | WFT AE |
