# Supplementary material for: A study of autoencoders as a feature extraction technique for spike sorting
Source: PLoS One. 2023 Mar 9;18(3):e0282810. doi: 10.1371/journal.pone.0282810 (PMC9997908; doi:10.1371/journal.pone.0282810)
Supplement: Attachment — Submitted filename: Autoencoders - Rebuttal Letter.docx [file pone.0282810.s008.docx]

Dear Editor(s),

We thank the reviewers for taking the time to read our manuscript and present their concerns. We have thoroughly revised the manuscript and addressed the points highlighted by the reviewers. We hope that the manuscript is suitable for publication now.

Response to the viewers:

## Reviewer #1

***Reviewer #1 Point 1*** *- This research failed to identify the problem and purpose of the study; hence, its novelty was minimal. In addition, the manuscript was poorly organized, thus I would recommend that the authors revise and resubmit it. In the event of resubmission, the following are some of my suggestions: Please adhere to the correct template, conduct additional literature reviews, compare your results to earlier work, and use standard citation format. Hope for the best.*

**Reply:** We have updated the manuscript structure such that it adheres to the submission guidelines, with modified section names and headings. With regard to the references, we have changed the Vancouver format to PLOS ONE’s version of Vancouver. Furthermore, we have restructured the paper to increase readability and clarity. We have added several explanations and literature citations to better illustrate the depth of our analysis. Moreover, we have extended the analysis of the methods from the 4 datasets described to all 95 synthetic datasets. The results are presented in the revised manuscript with the statistical evaluation using t-tests, with Bonferroni correction, shown in the Supplementary Material.

## Reviewer #2

***Reviewer #2 Point 1 -*** *Overall the work is interesting and useful. The proposed methods show promise. The evaluation methods are fairly rigorous, but can be improved by looking at dependence on parameter tuning and computing statistics. The presentation needs to provide some missing details. It was fun to read a paper from Transylvania on Halloween.*

**Reply:** First of all, we would like to thank the reviewer for the insightful comments. We struggled to address them as thorough as we could and performed a major revision of the manuscript. We have added the indicated parameter tuning of the classical feature extraction methods and have computed t-tests with a Bonferroni correction. These are now presented in the updated manuscript and the Supplementary Material document.

***Reviewer #2 Point 2 –*** *Line 57: If the authors want to be more specific here, the problem isn't so much an imbalance, (a known imbalanced distribution can be handled) but unknown distribution, since changing firing rates mean different relative frequencies at different times.*

**Reply:** We have added the suggested specifications in the updated manuscript.

***Reviewer #2 Point 3 –*** *Line 59: Perhaps the authors can discuss the concept of single units and multiunits here and state what they mean when they write neurons in the manuscript.*

**Reply:** We have added additional explanations on the concept of single and multi-units.

***Reviewer #2 Point 3 –*** *Line 61: Depending on the implementation, the 4th step is sometimes an offline (batch mode) clustering step, or alternatively an online classification step (using parameters previously found in offline batch mode clustering or by hand). This is a minor detail the authors can choose to include to be thorough.*

**Reply:** We included a short description of the suggested information.

***Reviewer #2 Point 4 –*** *Line* 100*: Around here would be a nice place for a brief discussion of why autoencoders are well suited for the spike sorting enterprise.*

**Reply:** We added a short discussion on why autoencoders would be a good fit for spike sorting at the indicated location.

***Reviewer #2 Point 5 –*** *Line* 156: *Consider mentioning wavelet transform (Yuan etal 10.1016/j.jneumeth.2012.07.012) and the traditional spike features like trough-to-peak height and trough to peak delay.*

**Reply:** We have added the suggested information in the ‘State of the Art’ section of the updated manuscript.

***Reviewer #2 Point 6 –*** *288: Is there a reason why numbering jumps from 4 to 16 to 35 (25?)*

**Reply:** The creators of these synthetic datasets created 95 of them. We have chosen four of these that we have found to be the most relevant based on the difficulties they provided. The number of clusters is incremented by four going from one dataset to another, such that it is becoming increasingly difficult to separate clusters as the overlap between them becomes larger. In the updated manuscript we now showcase an analysis over all 95 datasets and evaluated the significance of the results using t-tests with a Bonferroni correction. These can be found in Figure 5 of the updated manuscript and in figure 3 of the Supplementary Material.

***Reviewer #2 Point 7 –*** *Missing variable explanations*

*Line 363: Define what MaxRI and ExpectedRI are.*

*Line 375: Define quantitatively what the homogeneity variable means, and what the completeness variable means. How is beta set?*

*Line 388: Define s, d, maxR.*

*Line 394: Define n,k.*

*Line 401: Define a,b.*

**Reply:** We have aggregated all these points under the same umbrella as they refer to the same type of overlook. In the updated manuscript we have added descriptions to every variable used in the equations.

***Reviewer #2 Point 8 –*** *Line 501: Why does Tied AE perform so poorly in the 3 external clustering measures? Did k-means randomly give a bad clustering?*

**Reply:** It is not a random bad clustering, as we show that the variability of K-Means is almost nonexistent in figure 1 of the Supplementary Material but rather a bad clustering due to the distribution of the datasets. The clusters, although separated, will be labelled incorrectly by K-Means due to their shape. We have added this information in the Discussion section of the updated manuscript.

***Reviewer #2 Point 9 –*** *It is common practice to highlight in bold the rows that perform the best in each column. Be consistent with the usage of commas in the CHS column.*

**Reply:** We would like to thank the reviewer for highlighting this issue. We have amended the manuscript and corrected these inconsistencies.

***Reviewer #2 Point 10 –*** *Suggest some summary of the tables, perhaps using a rank aggregation method to give an overall ranking for each method (other methods are possible). Currently, many result values are presented but there are no overall statistics or significance testing (which may not be appropriate, but would be nice if this could be incorporated, say, by repeating simulations many times and analyzing the rankings of performance).*

**Reply:** We have added the ranking of the Borda rank aggregation algorithm for the synthetic datasets (table 1) and the real data (table 3) in the Supplementary Material document. Additionally, as we evaluated the algorithms on all synthetic datasets in the revised manuscript, we have added the ranking on all 95 synthetic datasets (table 2) in the Supplementary Material as well.

***Reviewer #2 Point 11 –*** *Line* 535*: For real data, please provide plots of the spike waveforms after clustering (clusters color coded or in subplots) for visual evaluation.*

**Reply:** We have added the requested evaluation in figure 8.

***Reviewer #2 Point 12 –*** *Line 599: While there are few parameters to tune (there are, in the learning algorithm for training the NN), the structure of the deep neural networks might be optimized per data set. Such tuning (and tuning of parameters for ICA and ISOMAP) may make a dramatic impact on the results. For maximum fairness, it would be best to at least do a cursory grid search over parameter values for the most sensitive parameters of each method; or show that the 6 metrics are not sensitive to parameter values (in supplementary). In anycase, all parameters used should be tabulated in the manuscript.*

**Reply:** As the architecture of the autoencoders and their parameters are the same over all datasets, we have considered it fair to have the same type of evaluation for the other methods as well. We have implemented a grid search over all the parameters of ICA and Isomap and changed the results with the best results found and the parametrization used for each dataset. Because for Isomap the impact of parameters on the performance metrics numbers in the thousands, we have not added all the results in the manuscript but updated the best and mentioned the value in the caption of the table. We have added the information about the grid search and the evaluated parameters at the beginning of the ‘Performance Evaluation’ section.

***Reviewer #2 Point 13 –*** *There is some randomness in the k-means clustering and NN training, so presenting the variability of the metrics in tables (in supplementary materials perhaps) would be useful.*

**Reply:** Indeed, we have added in the Supplementary Materials document figures that incorporate the variability of K-Means (figure 1) and the training of the autoencoder (figure 2).

Finally, we would like to thank the editor and the two reviewers for their insightful and helpful comments!
