# Supplementary material for: A study of autoencoders as a feature extraction technique for spike sorting
Source: PLoS One. 2023 Mar 9;18(3):e0282810. doi: 10.1371/journal.pone.0282810 (PMC9997908; doi:10.1371/journal.pone.0282810)
Supplement: Attachment — Submitted filename: Comments.docx [file pone.0282810.s009.docx]

A Study of Autoencoders as a Feature Extraction Technique for Spike Sorting

- Eugen Richard Ardelean

Introduction:

The introduction should describe the overview of your research, background, or brief of existing research, which methods you approached, research problems, and statements.

To write an introduction you can follow the following steps:

Introduce your topic:

Describe the background:

Establish your research problem:

The goal of your research:

Map of your paper:

Line 113-117: The description in this paragraph does not correlate with the subsections presented in the article. I could not connect your statement to sections iii, iv, and v (section: v does not exist at all).

Materials And Methods:

Line 88 & 201: Figure 1 & 2 “Code” Most research is commonly used as Latent Space.

Method:

Line 247-253: It will be nice if you write steps as numbering instead of bullet points.

Discussion:

Line 531-534: Could you please explain fig 4, which one is under clustering and over clustering? Which color shows which information?

Line 533: What is sim14?

Line 542: How and why did you choose the number of epochs? Please provide the references.

Result:

Line 552, 622, 639: This description should be in the table title. Please make the sentences as the title and discuss the performance and analysis of the comparison.

In tables 3, 4, 5, 6, 7, 8, and so on you said comparison but did not explain the comparison. Why did you bold .99, .98, .27 33.910, and others? What was the exact result of your research comparison?

Figures 6, and 7 you marked as a,b,c,d… but did not find any discussion about it.

Please compare your results with those of previous studies. Or there was no previous work such as this one?

Conclusion:

“Performance by comparing them to state-of-the-art algorithms” no comparison with other studies has been presented. Although, a comparison was made with conventional spike sorting techniques. This does not necessarily translate to state-of-the-art.

Final Comment:

Please accept my gratitude for the outstanding work you have done. More research on the topic at hand is strongly suggested. Your thesis should be written as a scholarly manuscript. In contrast to a research article, a thesis is written in a different format. I will suggest taking a look at the articles that were submitted before in the journal and the way the author organized their good work. Regarding future projects, you should conduct further literature reviews. We hope that your future writing ventures are as successful as your past ones have been.
