# Supplementary material for: A study of autoencoders as a feature extraction technique for spike sorting
Source: PLoS One. 2023 Mar 9;18(3):e0282810. doi: 10.1371/journal.pone.0282810 (PMC9997908; doi:10.1371/journal.pone.0282810)
Supplement: Attachment — Submitted filename: Autoencoders - Rebuttal Letter.docx [file pone.0282810.s010.docx]

Dear Editor(s),

We thank the reviewers for taking the time to read our manuscript and present their concerns. We have thoroughly revised the manuscript and addressed the points highlighted by the reviewers. We hope that the manuscript is suitable for publication now.

Response to reviewers:

## Reviewer #1

***Reviewer #1 Point 1 –*** *Introduction*

*The introduction should describe the overview of your research, background, or brief of existing research, which methods you approached, research problems, and statements.*

*To write an introduction you can follow the following steps:*

*Introduce your topic:*

*Describe the background:*

*Establish your research problem:*

*The goal of your research:*

*Map of your paper:*

**Reply:** We have taken into account the points of all reviewers and we have now reformatted the introduction according to their suggestion, to the best of our ability. It is now separated into three subsections. Section 1.1 introduces the topic of spike sorting—the main topic of the paper. Section 1.2 cursory describes autoencoders and outlines the necessary background. Finally, section 1.3 establishes the challenges of spike sorting that define our research problem and states the goal of the research. As suggested by the reviewer, the introduction ends with a map of the paper.

***Reviewer #1 Point 2 –*** *Line 113-117: The description in this paragraph does not correlate with the subsections presented in the article. I could not connect your statement to sections iii, iv, and v (section: v does not exist at all).*

**Reply:** A major restructuring of the manuscript has been made in a previous stage of revision and we overlooked modifying this paragraph. We would like to thank the reviewers for point it out for us. We have now fixed this.

***Reviewer #1 Point 3 –*** *Materials And Methods:*

*Line 88 & 201: Figure 1 & 2 “Code” Most research is commonly used as Latent Space.*

**Reply:** The figures have been modified such that instead of ‘Code’, they now contain ‘Latent space’.

***Reviewer #1 Point 3 –*** *Method:*

*Line 247-253: It will be nice if you write steps as numbering instead of bullet points.*

**Reply:** The steps of pretraining have been modified from bullet points to numbering.

***Reviewer #1 Point 4 –*** *Discussion:*

*Line 531-534: Could you please explain fig 4, which one is under clustering and over clustering? Which color shows which information?*

**Reply:** Indeed, we have not explained this properly. In case of fig 4, neither of them shows either under or overclustering, because the colors display the ground truth and not the result of a clustering algorithm. Under and overclustering are generated by clustering algorithms in specific cases, when compared to a ground truth. When two (or more) clusters in the ground truth are labelled as one by the clustering algorithm, it is considered underclustering. While, when a single cluster in the ground truth is considered as two (or more) clusters by the clustering algorithm, we can say that the algorithm is overclustering. Additional explanations have now been added to the manuscript.

***Reviewer #1 Point 5 –*** *Line 533: What is sim14?*

**Reply:** Simulation 14 is another dataset that is encompassed in the 95 synthetic datasets used [45]. We have chosen to show the impact of alignment on simulation 14 because it has a lower number of clusters and samples and is therefore easier to visualize. We have added additional information about it, where we also discuss the other simulations, in section 2.3.1.

***Reviewer #1 Point 6 –*** *Line 542: How and why did you choose the number of epochs? Please provide the references.*

**Reply:** In table 2, we show the impact of the number of epochs (through a selected number of samples) on the performance of the model with regard to the metrics. We have chosen the number of epochs based on the results of the metrics and the loss of the model. We add below a plot of the loss values across epochs, showing that at around 50 epochs the loss of model saturates and increasing the number of epochs would not bring significant benefit. This figure has also been added to the Supporting Information and is now referenced in the manuscript.

***Reviewer #1 Point 7 –*** *Result:*

*Line 552, 622, 639: This description should be in the table title. Please make the sentences as the title and discuss the performance and analysis of the comparison.*

**Reply:** The indicated lines have been modified (and explanations have been added) according to the suggestions of the reviewer.

***Reviewer #1 Point 8 –*** *In tables 3, 4, 5, 6, 7, 8, and so on you said comparison but did not explain the comparison. Why did you bold .99, .98, .27 33.910, and others? What was the exact result of your research comparison?*

**Reply:** The caption of each table has been modified according to the suggestion at the previous point. The bolded values highlight the best values for each metric (we say best instead of highest because the DBS metric is inverse, with lower values indicating better clustering), thus indicating the best performing methods according to each metric. We now mention this in the caption, for each table. The overall conclusions that can be extracted from the analysis are now discussed in section 4.

***Reviewer #1 Point 9 –*** *Figures 6, and 7 you marked as a,b,c,d… but did not find any discussion about it.*

**Reply:** The reviewer is right, this was a mistake. Figures 6 and 7 now no longer contain any alphabetical labelling, as this is not necessary and not used.

***Reviewer #1 Point 10 –*** *Please compare your results with those of previous studies. Or there was no previous work such as this one?*

**Reply:** We have referenced two recent papers that study autoencoders in spike sorting ([15] and [16]). We have now described their results in the introduction. However, one major issue preventing a direct comparison to our approach is that these previous studies have been limited to synthetic datasets that contain a low number of clusters and, in addition, their evaluation was restricted to accuracy for [15] and, accuracy and silhouette score for [16]. We already discussed that accuracy is not a suitable metric for the problem of spike sorting because: i) it can only be used for synthetic datasets that contain a ground truth; and ii) for datasets that have a low amount of imbalance. Imbalance is an inherent problem in spike sorting because different neurons usually have very different firing rates, thus generating clusters of different densities. The typical imbalance observed in spike datasets renders accuracy quite problematic as a performance metric. We now elaborate on these concerns when discussing performance metrics in the manuscript. In [16], the authors propose contractive autoencoders for spike sorting, which is one of the variants that we also evaluate. Thus, we now mention the previous studies we could find, we discuss their results, but also point out the limitations in relating to their results due to the use of accuracy as a performance metric on imbalanced data.

***Reviewer #1 Point 11 –*** *Conclusion:*

*“Performance by comparing them to state-of-the-art algorithms” no comparison with other studies has been presented. Although, a comparison was made with conventional spike sorting techniques. This does not necessarily translate to state-of-the-art.*

**Reply:** We have now reformulated this part of the conclusions.

## Reviewer #2

*The authors have addressed all of my concerns and the manuscript is much improved.*

*The authors may wish to pay attention to legibility of words in figures during proof, as the words were very small in the review pdf.*

**Reply:** We would like to thank the reviewer for the insightful comments in the previous stage of revision.

Finally, we would like to thank the editor and the two reviewers for their insightful and helpful comments!
